# Effects of Probiotics on Colitis-Induced Exacerbation of Alzheimer’s Disease in *App^NL-G-F^* Mice

**DOI:** 10.3390/ijms241411551

**Published:** 2023-07-17

**Authors:** Bijayani Sahu, Lauren M. Johnson, Mona Sohrabi, Anastasia A. Usatii, Rachel M. J. Craig, Joshua B. Kaelberer, Sathiya Priya Chandrasekaran, Harpreet Kaur, Suba Nookala, Colin K. Combs

**Affiliations:** 1 Department of Biomedical Sciences, School of Medicine and Health Sciences, University of North Dakota, Grand Forks, ND 58202, USA; bijayani.sahu@und.edu (B.S.); lauren.johnson.6@und.edu (L.M.J.); mona.sohrabi@und.edu (M.S.); usati002@umn.edu (A.A.U.); rmc288@cornell.edu (R.M.J.C.); joshua.kaelberer@und.edu (J.B.K.); s.chandrasekaran@und.edu (S.P.C.); suba.nookala@und.edu (S.N.); 2 The Jackson Laboratory, Bar Harbor, ME 04609, USA; harpreet.kaur@jax.org

**Keywords:** Alzheimer’s, colitis, intestine, amyloid

## Abstract

Alzheimer’s disease (AD) is characterized by progressive cognitive decline and is a leading cause of death in the United States. Neuroinflammation has been implicated in the progression of AD, and several recent studies suggest that peripheral immune dysfunction may influence the disease. Continuing evidence indicates that intestinal dysbiosis is an attribute of AD, and inflammatory bowel disease (IBD) has been shown to aggravate cognitive impairment. Previously, we separately demonstrated that an IBD-like condition exacerbates AD-related changes in the brains of the *App^NL-G-F^* mouse model of AD, while probiotic intervention has an attenuating effect. In this study, we investigated the combination of a dietary probiotic and an IBD-like condition for effects on the brains of mice. Male C57BL/6 wild type (WT) and *App^NL-G-F^* mice were randomly divided into four groups: vehicle control, oral probiotic, dextran sulfate sodium (DSS), and DSS given with probiotics. As anticipated, probiotic treatment attenuated the DSS-induced colitis disease activity index in WT and *App^NL-G-F^* mice. Although probiotic feeding significantly attenuated the DSS-mediated increase in WT colonic lipocalin levels, it was less protective in the *App^NL-G-F^* DSS-treated group. In parallel with the intestinal changes, combined probiotic and DSS treatment increased microglial, neutrophil elastase, and 5hmC immunoreactivity while decreasing c-Fos staining compared to DSS treatment alone in the brains of WT mice. Although less abundant, probiotic combined with DSS treatment demonstrated a few similar changes in *App^NL-G-F^* brains with increased microglial and decreased c-Fos immunoreactivity in addition to a slight increase in Aβ plaque staining. Both probiotic and DSS treatment also altered the levels of several cytokines in WT and *App^NL-G-F^* brains, with a unique increase in the levels of TNFα and IL-2 being observed in only *App^NL-G-F^* mice following combined DSS and probiotic treatment. Our data indicate that, while dietary probiotic intervention provides protection against the colitis-like condition, it also influences numerous glial, cytokine, and neuronal changes in the brain that may regulate brain function and the progression of AD.

## 1. Introduction

Alzheimer’s disease (AD) is a neurodegenerative disease that gradually deteriorates behavioral and cognitive functions such as memory, comprehension, language, attention, reasoning, and judgment [1,2,3]. Approximately 57.4 million individuals worldwide had dementia in 2019, and by 2050, that number is expected to rise to 152.8 million [4]. With an estimated 6.5 million cases in the USA and an expected increase to 13.8 million cases by 2060, AD is the seventh leading cause of death [2,5]. Its main pathological features are the deposit of β-amyloid (Aβ) [6] peptides in the extracellular space and the formation of neurofibrillary tangles [7,8,9] arising from the intraneuronal accumulation of hyperphosphorylated tau protein [10]. Unfortunately, the current treatments for Alzheimer’s disease provide only marginal benefits [11,12]. Furthermore, many highly promising drugs have failed to demonstrate clinical benefits in phase III trials [13]. Even recent results from anti-Aβ immunotherapy are not entirely clear, suggesting that additional data are required to confirm its efficacy [11,12,14]. Therefore, due to an increased need to better understand the disease and its comorbidities, developing therapies that alter disease progression remains a priority.

Recent studies, including experimental and clinical evidence, have suggested that gut dysbiosis and gut microbiota–host interactions play an important role in neurodegeneration [15,16,17,18,19,20]. The combination of a gut-derived inflammatory response, aging, and a poor diet in the elderly may all contribute to the pathophysiology of AD. Alteration of the gut microbiota composition through food-based therapy or probiotic supplementations may open new preventive and therapeutic avenues in AD. There is also promising evidence that the intestinal microbiota influences brain–gut interactions at various ages and at various levels in the intestine [21]. For example, animal studies using germ-free mice show that gut microbiota play an important role in early brain development and adult neurogenesis [19,21,22]. The impact of microbiota on the brain through the so-called brain–gut-microbiota axis is mediated by neural, immune, endocrine, and metabolic signaling [22].

Irritable bowel disease, IBD, primarily consists of ulcerative colitis (UC) and Crohn’s disease (CD) and is a chronic inflammatory condition marked by alternating periods of disease activity and latency [23,24,25]. It is characterized by inflammation, which can cause abdominal pain, diarrhea, and bloody stool [26]. Interestingly, the link between chronic inflammation and cognitive decline has been reported in several studies [27,28,29]. Systemic inflammation may drive neuroinflammatory changes and chronic activation of microglia, leading to oxidative stress and the deposition of misfolded proteins in Alzheimer’s dementia [30]. In addition, there is promising evidence of gut-derived products serving as a pathogenic link between immune activation and AD [31]. Thus, there is a convincing biological possibility for a predisposition of AD in IBD patients [31,32,33,34]. 

Previously, we demonstrated that an IBD-like condition induced by DSS exacerbates AD-related changes in the brains of *App^NL-G-F^* mice [35]. In this study, we investigated whether dietary intervention with a probiotic could attenuate IBD-potentiated changes in the brains of these mice. We employed a commercially available probiotic cocktail composed of eight strains of lactic acid-producing bacteria: *Lactobacillus plantarum*, *Lactobacillus delbrueckii* subsp. *Bulgaricus*, *Lactobacillus paracasei*, *Lactobacillus acidophilus*, *Bifidobacterium breve*, *Bifidobacterium longum*, *Bifidobacterium infantis*, and *Streptococcus salivarius* subsp. *Thermophilus* [36].

## 2. Results

### 2.1. Probiotic Ameliorated the DSS-Induced Colitis-like Condition in the Intestine

**Figure 1:**
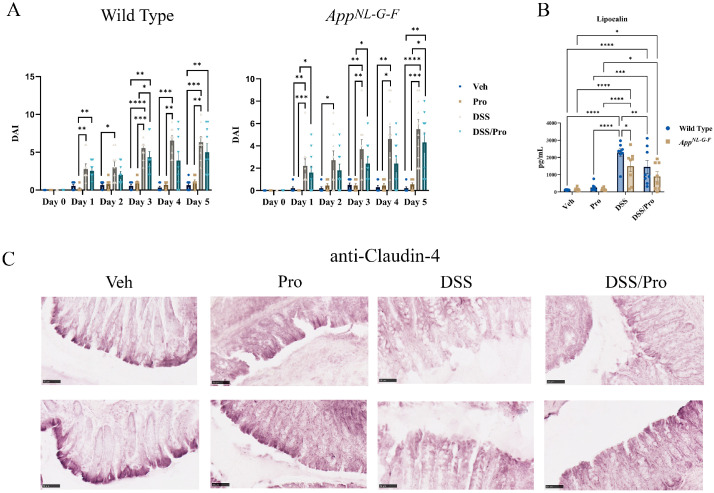
**Probiotic treatment had minimal effects on DSS-induced colitis disease activity index (DAI), lipocalin levels, and claudin-4 immunoreactivity in C57BL/6J wild type (WT) and *App^NL-G-F^* mice.** Male C57BL/6J wild type and *App^NL-G-F^* mice were given diluted MediGel in water (1:1) ad libitum or the probiotic resuspended in diluted MediGel starting at week 0 and until week 8. At weeks 3 and 5, DSS was provided in the diluted MediGel (2% final concentration) for three days per cycle. Mice were allowed to recover until week 8 with or without maintained exposure to the probiotic. (**A**) A colitis-like disease (DAI) was assessed in vehicle, probiotic (Pro), DSS, and DSS/Pro treatment groups in male wild type and *App^NL-G-F^* mice. The DAI was monitored on the second 3-day cycle of 2% DSS exposure and 2 days afterwards (day 0–day 5) in all treatment groups. (**B**) Upon completion of the probiotic feeding paradigm at 8 weeks, the wild type and *App^NL-G-F^* mice were collected before colon lipocalin levels were quantified by ELISA. (**C**) Claudin 4 immunoreactivity was examined in colons of both wild type and *App^NL-G-F^* colons using Vector VIP as the chromogen. Representative images are shown (scale bar 50 μm). Non-parametric one-way ANOVA (Kruskal–Wallis test) followed by Dunn’s multiple comparisons test was used to determine statistical differences. Results are presented as mean ± SEM, * *p* < 0.05, ** *p* < 0.01, *** *p* < 0.001, and **** *p* < 0.0001.

Male *App^NL-G-F^* and WT mice were fed probiotic ad libitum for three weeks prior to treatment with 2% DSS for two bouts of three days each with a fourteen-day interval in between to model two episodes of colonic inflammation. Mice remained on probiotic feeding throughout the entire experimental period. The symptomatic parameters of colitis, disease activity index (DAI), colonic lipocalin levels, and claudin 4 immunoreactivity were assessed after the second round of DSS treatment. The DAI includes an assessment of stool consistency, occult fecal blood, and percent body weight loss and is associated with colonic inflammation and the presence of gut lesions. Each parameter was rated on a scale of 0–4 and then scored out of 12, indicating the maximum DAI for each condition. This method of scoring is similar to the clinical presentation of IBD symptoms in humans. During the second administration of DSS, both WT and *App^NL-G-F^* mice displayed significantly greater disease activity scores compared to their respective untreated groups (Figure 1A). This increase was reduced in both genotypes at day four by probiotic feeding (Figure 1A). To examine the more long-term recovery benefits of probiotic feeding, the mice remained on the probiotic diet until the eighth week of the experiment. Fecal lipocalin-2 is a stable, highly sensitive, and non-invasive marker that determines the extent of intestinal inflammation [37]. Colonic lipocalin levels were significantly increased even at 9 weeks in response to DSS treatment in *App^NL-G-F^* and WT mice compared to their controls (Figure 1B). However, the probiotic feeding reduced lipocalin levels in the WT mice, indicating a recovery benefit that was not observed in *App^NL-G-F^* mice (Figure 1B). Increasing evidence from studies involving IBD patients and animal models suggests that the downregulation or redistribution of claudins is strongly involved in the pathogenesis of IBD, including colitis [38,39,40,41]. Among these, it has been reported that the sealing tight junction protein claudin-4 functions as a paracellular sodium barrier and that the downregulation of claudin-4 expression could decrease transepithelial electrical resistance (TER) [42]. To assess the colonic epithelial integrity, immunohistochemistry staining for claudin-4 was performed. The DSS-treated groups in both WT and *App^NL-G-F^* mice showed reduced staining for claudin-4, which remained low with probiotic feeding, in alignment with the maintained inflammation demonstrated by the lipocalin ELISA results (Figure 1C). Interestingly probiotic feeding itself appeared to reduce claudin-4 immunoreactivity (Figure 1C).

### 2.2. DSS-Induced Colitis and Probiotic Administration Increased Neutrophil Elastase Immunoreactivity in the Brain

**Figure 2:**
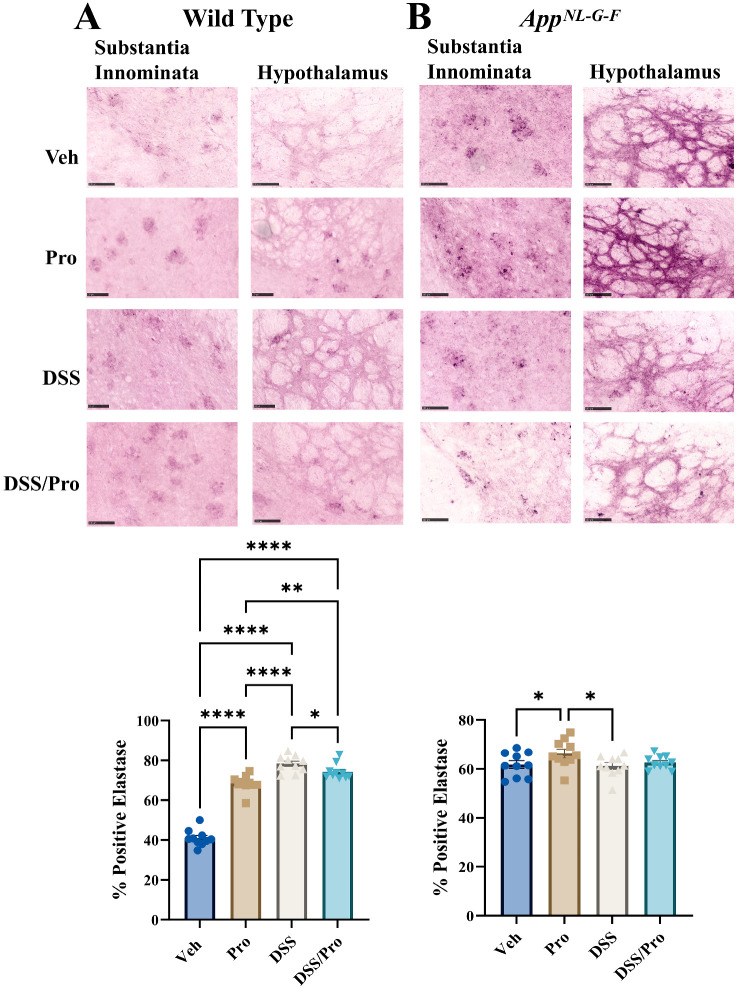
**DSS and probiotic increased brain neutrophil elastase immunoreactivity in wild type but not *App^NL-G-F^* mice.** After the 8 weeks of probiotic feeding, brains of (**A**) wild type and (**B**) *App^NL-G-F^* mice were fixed and serial sectioned (40 µm) for anti-neutrophil elastase immunohistochemistry. Representative neutrophil elastase immunohistochemical staining images (20×) of the substantia innominata and hypothalamus are shown (scale bar: 100 µm). Elastase positive cell counts were quantified from an entire hemibrain coronal section from 3 to 10 sections per brain in each group. The positive counts were then measured as a percentage of the annotated area. One-way ANOVA followed by uncorrected Fisher’s LSD test was used to determine statistical differences. Results are presented as mean values + SEM, * p < 0.05, ** p < 0.01, and **** p < 0.0001.

In colitis, it has been established that there is an infiltration of neutrophils, and their activation results in the excessive release of neutrophil elastase, which is implicated in colon inflammation and severe colitis [43]. To assess the possible gut–brain communication of the colitis-associated immune changes, we elected to examine the possible infiltration of the brain by neutrophils by performing immunohistochemical analysis for neutrophil elastase. Interestingly, when quantifying overall hemibrain coronal section immunoreactivity, both probiotic and DSS treatment increased elastase immunoreactivity in WT brains compared to the vehicle controls, while only probiotic increased immunoreactivity in *App^NL-G-F^* brains (Figure 2). Greater inspection of the immunohistochemical changes demonstrated a few areas with particularly robust elastase immunoreactivity. For example, staining was observed in the substantia innominata in both WT and *App^NL-G-F^* brains (Figure 2). The substantia innominata contains the nucleus basalis of Meynert, a brain region responsible for producing the acetylcholine that is used by the cortex and amygdala [44]. The degeneration of these neurons contributes to the cholinergic deficit observed in AD [45]. The substantia innominata also has a role in the regulation of aggressive behaviors due to its association with the amygdala and midbrain [46]. Interestingly, all groups in the WT and *App^NL-G-F^* mice also demonstrated a netted “web-like” neutrophil elastase immunoreactivity pattern in the hypothalamus (Figure 2), suggesting the possibility of neutrophils undergoing netosis within this region [47].

### 2.3. Probiotic and DSS-Mediated Alterations in Brain Cytokines

**Figure 3:**
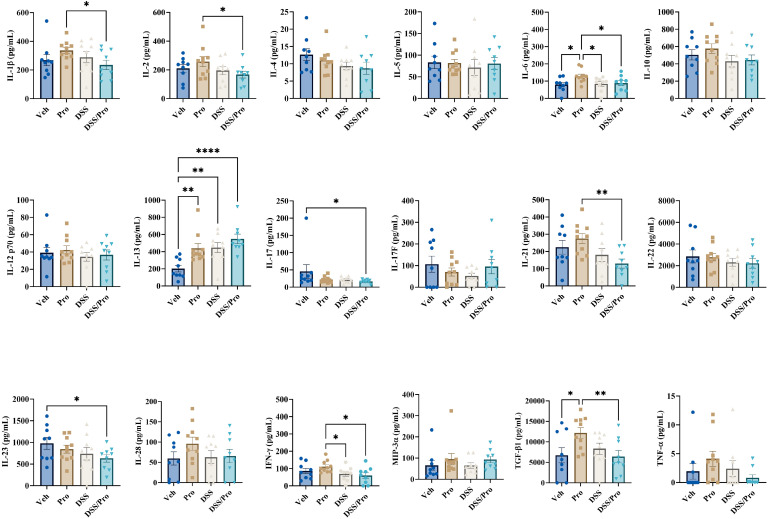
**DSS and probiotic altered numerous cytokine levels in the cortices of wild type mice.** After completing the probiotic feeding paradigm at 8 weeks, C57BL/6J wild type mice were collected, and temporal cortex cytokine levels were quantified by commercial slide array. One-way ANOVA followed by uncorrected Fisher’s LSD test was used to determine statistical differences. Results are presented as mean ± SEM, * *p* < 0.05, ** *p* < 0.01, and **** *p* < 0.0001.

**Figure 4:**
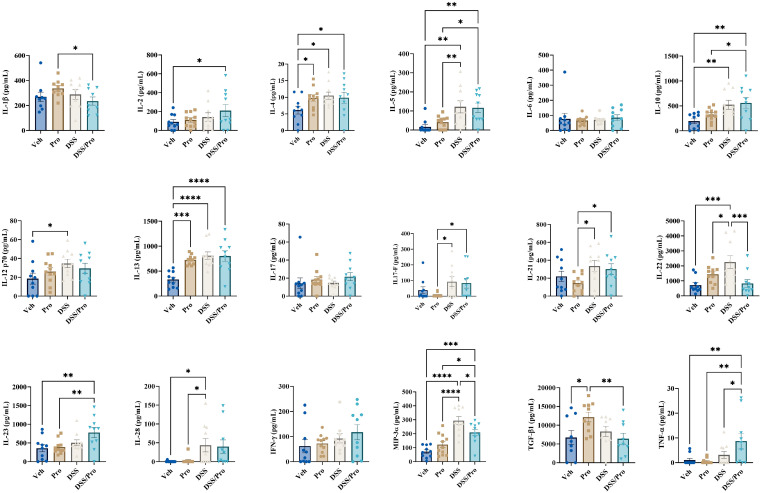
**DSS and probiotic altered numerous cytokine levels in the cortices of *App^NL-G-F^* mice.** After completing the probiotic feeding paradigm at 8 weeks, *App^NL-G-F^* mice were collected, and temporal cortex cytokine levels were quantified by commercial slide array. One-way ANOVA followed by uncorrected Fisher’s LSD test was used to determine statistical differences. Results are presented as mean ± SEM, * *p* < 0.05, ** *p* < 0.01, *** *p* < 0.001, and **** *p* < 0.0001.

To further assess the neuroinflammatory status induced by DSS treatment with or without the probiotic intervention, a slide-based cytokine array was performed on lysates from the temporal cortices of WT and *App^NL-G-F^* mice. As shown in Figure 3, compared to vehicle controls, all the three treatments significantly elevated IL-13 levels in the WT brain cortices. In addition to IL-13, probiotic feeding significantly upregulated the cortical levels of IL-6 and TGF-β1 in the WT mice. Interestingly, combined DSS+Pro treatment significantly attenuated the cortical levels of probiotic-induced pro-inflammatory mediators IL-1β, IL-2, IL-6, IL-21, and IFN-γ, and the regulatory TGF-β1 in the brains of the WT mice (Figure 3). Furthermore, combined DSS+Pro treatment in WT mice resulted in a significant reduction in cortical IL-17 levels compared to vehicle controls (Figure 3). We observed a strikingly different cortical cytokine profile in the vehicle and treatment groups of *App^NL-G-F^* mice. Interestingly, all the three treatments induced Th2 family cytokines, IL-4 and IL-13, compared to the vehicle controls (Figure 4). In addition to IL-4 and IL-13, probiotic feeding significantly upregulated the cortical levels of TGF-β1 in *App^NL-G-F^* mice (Figure 4). The influence of DSS treatment on cortical cytokines was readily noticeable in *App^NL-G-F^* mice. Compared to vehicle controls, treatment with DSS alone also significantly increased the cortical levels of IL-10, IL-12p70, IL-22, IL-28, and MIP-3α (Figure 4). Interestingly, combined DSS and probiotic treatment showed both alleviating and intensifying effects on probiotic-induced cortical cytokine levels in *App^NL-G-F^* mice. Our data show that, while DSS and probiotic treatment significantly attenuated the cortical levels of probiotic-induced IL-1β, IL-22, and TGF-β1 (Figure 4), an opposite effect was apparent, with a significant increase in the probiotic-induced cortical levels of IL-5, IL-10, IL-17F, IL21, IL-23, MIP-3α, and TNF-α (Figure 4). Interestingly, combined DSS and probiotic treatment uniquely increased IL-2 and TNF-α from vehicle controls, demonstrating an additional effect of the dietary intervention (Figure 4).

### 2.4. DSS- and Probiotic-Mediated Alterations in Hippocampal Aβ Accumulation

**Figure 5:**
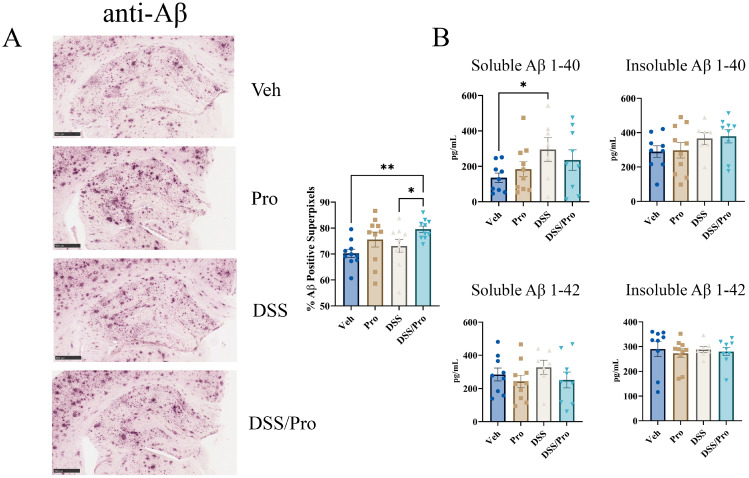
**DSS increased hippocampal Aβ levels in *App^NL-G-F^* mouse brains.** After completing the probiotic feeding paradigm at 8 weeks, wild type and *App^NL-G-F^* mice were collected. (**A**) Left hemispheres of *App^NL-G-F^* mice were fixed and serial sectioned (40 µm) for anti-Aβ immunohistochemistry. Percent Aβ positive super pixels in the hippocampus were determined from three sections per mouse in each condition. One-way ANOVA followed by uncorrected Fisher’s LSD test was used to determine statistical differences. Representative 5× images are shown (scale bar 500 μm). Results are presented as mean ± SEM, * *p* < 0.05, ** *p* < 0.01 (n = 10). (**B**) Hippocampal levels of human-soluble and insoluble Aβ 1-40 and 1-42 were quantified by ELISA from *App^NL-G-F^* lysates. One-way ANOVA followed by uncorrected Fisher’s LSD test was used to determine statistical differences. Results are presented as mean ± SEM, * *p* < 0.05 and ** *p* < 0.01.

Since Aβ plaque accumulation is a key pathological finding in AD brains [2], we subsequently elected to explore whether probiotic and/or DSS treatment altered brain Aβ plaque load in *App^NL-G-F^* mice. Surprisingly, Aβ immunohistochemistry demonstrated a slight increase in *App^NL-G-F^* brain plaque load following combined DSS and probiotic treatment compared to the vehicle controls (Figure 5). This Aβ immunoreactivity was further validated by performing ELISAs on hippocampal lysates to quantify soluble and insoluble Aβ 1-40 and Aβ 1-42. Interestingly, DSS increased soluble Aβ 1-40 concentrations compared to vehicle controls, although neither treatment alone produced significant differences compared to the vehicle group for either peptide (Figure 5).

### 2.5. DSS- and Probiotic-Associated Alterations in Hippocampal Gliosis

**Figure 6:**
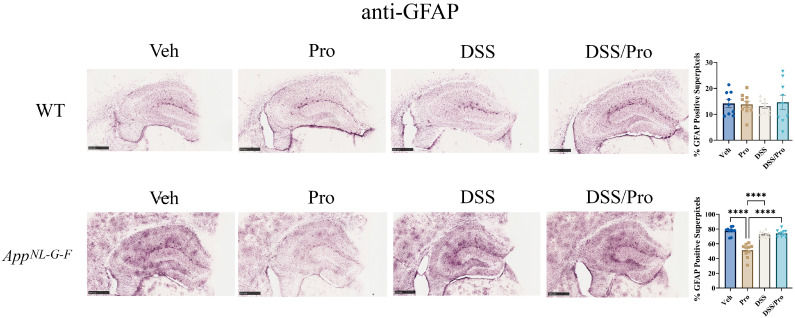
**Probiotic treatment reduced astrogliosis in the hippocampus of *App^NL-G-F^* mice.** After 8 weeks of probiotic feeding, the brains of the *App^NL-G-F^* mice and wild type mice were fixed and serial sectioned (40 µm) for anti-GFAP immunohistochemistry. The percentage of GFAP-positive super pixels in the hippocampus were determined from three sections per mouse in each condition. One-way ANOVA followed by uncorrected Fisher’s LSD test was used to determine statistical differences. Results are presented as mean ± SEM; **** *p* < 0.0001. Representative 5X images are shown (scale bar: 500 μm).

Astrocytes are the most abundant glial subtype in the CNS; they play a crucial role in the regulation of neuroinflammation, and there are several reports that suggest they are associated with the senile plaques in the brains of AD patients [48,49,50,51]. To investigate whether probiotic and/or DSS treatment altered astrogliosis, brain sections were immunostained for the glial acidic fibrillary protein (GFAP). In WT mice, a basal level of GFAP staining was detected, which was not altered by either the DSS or probiotic treatment (Figure 6). In *App^NL-G-F^* mice, probiotic treatment alone reduced GFAP immunoreactivity compared to the vehicle control group, although there was no effect in the other treatment groups (Figure 6).

**Figure 7:**
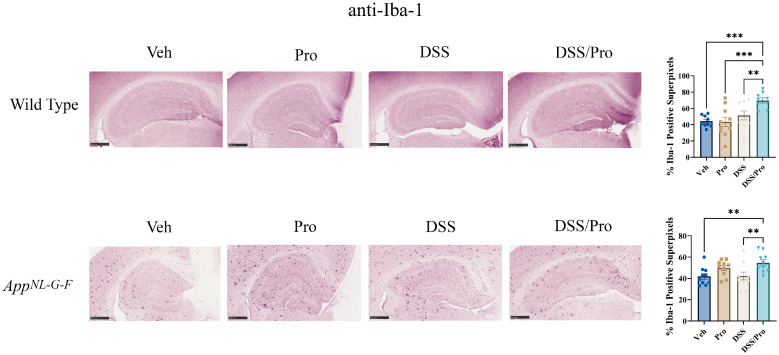
**Combined DSS and probiotic treatment increased microgliosis in the hippocampus of wild type and *App^NL-G-F^* mice.** After the 8 weeks of probiotic feeding, the brains of *App^NL-G-F^* and wild type mice were fixed and serial sectioned (40 µm) for anti-Iba-1 immunohistochemistry. The percentage of Iba-1-positive super pixels in the hippocampus were determined from three sections per mouse in each condition. One-way ANOVA followed by uncorrected Fisher’s LSD test was used to determine statistical differences. Results are presented as mean ± SEM, ** *p* < 0.01, and *** *p* < 0.001. Representative 5× images are shown (scale bar: 500 μm).

Microglial activation is also hypothesized to have a role in AD pathophysiology [52,53,54,55]. To examine the effect of DSS-induced colitis and the probiotic treatments on microglial reactivity, mouse brain sections were immunostained for the ionized calcium-binding adapter molecule 1 (Iba1) protein. The basal level of immunoreactivity observed in WT mice was not altered by either probiotic or DSS treatment (Figure 7). However, combined probiotic and DSS treatment actually significantly increased Iba1 immunoreactivity in WT mice compared to the vehicle control group, suggesting a combined effect in the brain (Figure 7). *App^NL-G-F^* mice displayed the characteristically robust Iba1 immunostaining associated with Aβ plaque accumulation (Figure 7). Just like in WT mice, combined DSS and probiotic treatments elevated Iba-1 immunoreactivity compared to AppNL-G-F vehicle controls (Figure 7).

### 2.6. DSS- and Probiotic-Associated Alterations in c-Fos Immunoreactivity

**Figure 8:**
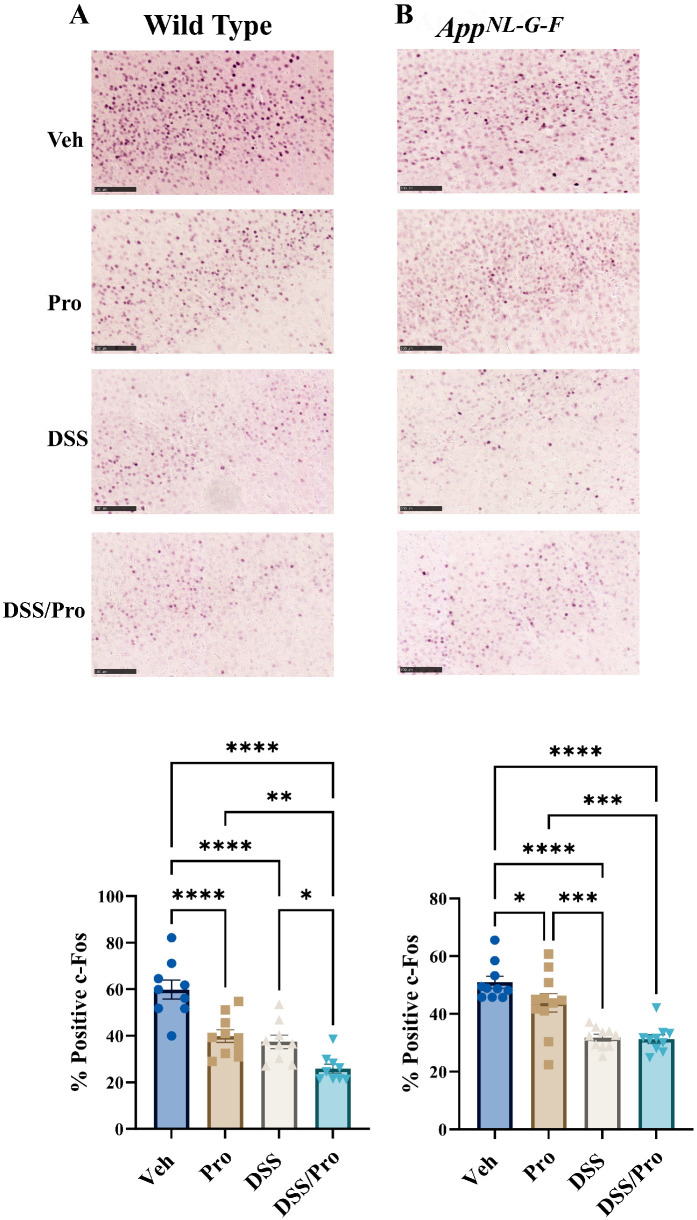
**DSS and probiotic attenuated c-Fos staining in the brains of wild type and *App^NL-G-F^* mice.** After 8 weeks of probiotic feeding, brains of (**A**) wild type and (**B**) *App^NL-G-F^* mice were fixed and serial sectioned (40 µm) for anti-c-Fos immunohistochemistry. Representative images (20×) from the parietal cortex are shown (scale bar: 100 μm). c-Fos-positive cell counts were quantified from an entire hemibrain coronal section from 3 to 10 sections per brain in each group. The positive counts were then measured as a percentage of the annotated area. Values are presented as mean ± SEM. One-way ANOVA followed by uncorrected Fisher’s LSD test was used to determine statistical differences; * *p* < 0.5, ** *p* < 0.01, *** *p* < 0.001, and **** *p* < 0.0001.

The transcription factor c-Fos is a well characterized immediate early gene in neurons, and its expression can be used as a surrogate of neuronal activity or phenotype changes [56,57]. Based upon the changes in glial reactivity, which were examined following DSS and probiotic treatment, we subsequently examined the changes in neuronal activation by quantifying c-Fos immunoreactivity in the brain sections of the mice. DSS treatment attenuated overall c-Fos staining in WT and *AppN^L-G-F^* brains compared to their respective vehicle controls in all three treatment groups, suggesting altered transcription in all conditions (Figure 8). Combined DSS and probiotic treatment further reduced c-Fos immunoreactivity compared to the groups treated with DSS or probiotic alone in WT mice (Figure 8).

### 2.7. DSS- and Probiotic-Associated Alterations in 5hmC Immunoreactivity

**Figure 9:**
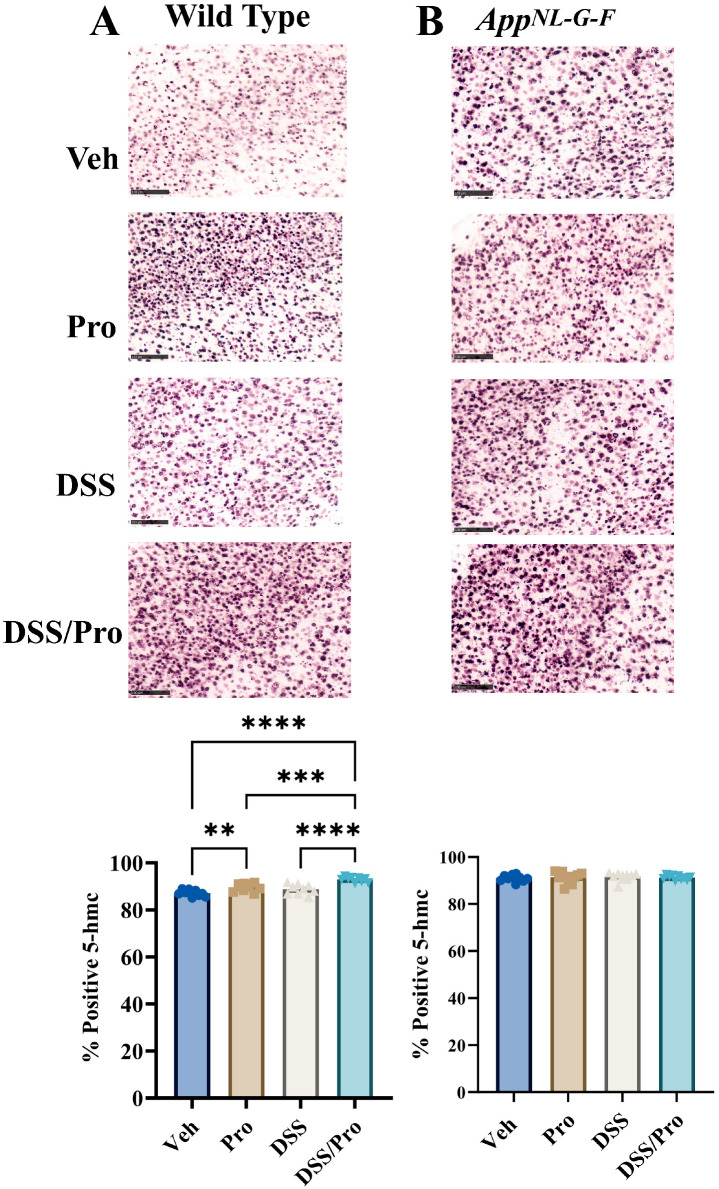
**Probiotic and DSS increased 5hmC staining only in the brains of wild type mice.** After 8 weeks of probiotic feeding, brains of (**A**) wild type and (**B**) *App^NL-G-F^* mice were fixed and serial sectioned (40 µm) for anti-5hmC immunohistochemistry. Representative images (20×) from the parietal cortex are shown (scale bar 100 μm). 5hmC positive cell counts were quantified from an entire hemibrain coronal section from 3 to 10 sections per brain in each group. The positive counts were then measured as a percentage of the annotated area. Values are presented as mean ± SEM. One-way ANOVA followed by uncorrected Fisher’s LSD test was used to determine statistical differences; ** *p* < 0.01, *** *p* < 0.001, and **** *p* < 0.0001.

As another indicator of possible changes in cellular phenotypes due to either DSS treatment or probiotic intervention, we subsequently immunostained the brain sections of the mice to detect the DNA modification 5-hydroxymethylcytosine (5hmC). 5hmC is generated from the oxidation of 5-methylcytosine (5mC) and is highly expressed in neurons in the brain [58,59]. Moreover, increased 5hmC is a useful indicator of gene demethylation and possible expression changes in neurons [60]. Probiotic and DSS and probiotic treatment groups increased overall hemibrain 5hmC immunoreactivity in WT brains compared to vehicle controls (Figure 9). Surprisingly, no changes in 5hmC immunoreactivity were observed in any treatment group of *App^NL-G-F^* mice, suggesting a resistance to this functional demethylation (Figure 9).

### 2.8. DSS- and Probiotic-Associated Alterations in Synaptic Proteins

**Figure 10:**
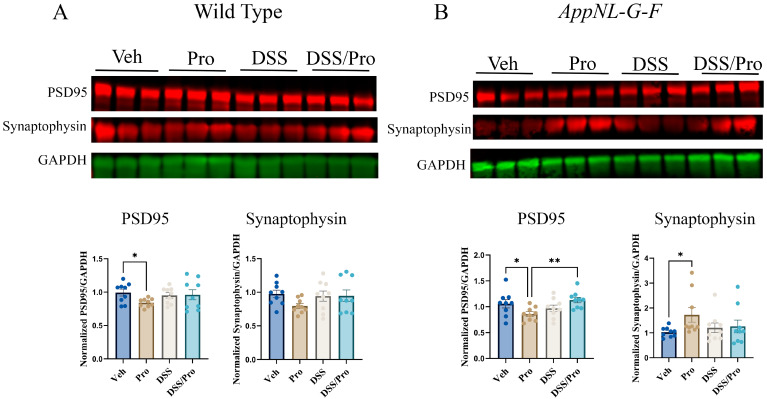
**DSS and probiotic effects on presynaptic or postsynaptic protein levels in the brains of wild type and *App^NL-G-F^* mice.** After 8 weeks of probiotic feeding, the temporal cortices of (**A**) wild type and (**B**) *App^NL-G-F^* mice treated with or without DSS were lysed, and the proteins were resolved by SDS-PAGE for Western blot analysis using antibodies against PSD95, synaptophysin, and GAPDH (loading control). Data from Western blots are graphed as mean ± SEM of PSD95 or synaptophysin values normalized to their respective GAPDH. One-way ANOVA followed by uncorrected Fisher’s LSD test was used to determine statistical differences; * *p* < 0.05 and ** *p* < 0.001.

As a final assessment of colitis- and probiotic-mediated changes in the brain, we examined the protein levels of post synaptic density protein 95, (PSD95) and synaptophysin to quantify postsynaptic and presynaptic compartment integrity, respectively. Although no overall dramatic changes were induced by DSS treatment in either WT or *App^NL-G-F^* mice, a slight decrease in PSD95 levels was noted compared to the vehicle control group following probiotic feeding in both lines (Figure 10). Probiotic feeding increased synaptophysin levels in *App^NL-G-F^* but not in WT mice (Figure 10).

## 3. Discussion

In this study, we investigated the effect of probiotics on the exacerbation of Alzheimer’s disease induced by chronic colitis. We have previously reported that colonic inflammation correlates with brain Aβ plaque deposition starting at 3 months of age in *App^NL-G-F^* mice and increased pro-inflammatory markers and macrophages in the ileums of *App^NL-G-F^* and APP/PS1 mice compared to controls and chronic intestinal disruption induced by two cycles of DSS exposure resulted in moderate colitis-like symptoms in WT and *App^NL-G-F^* animals [35]. In this study, we again used a DSS-induced colitis model, which is characterized by intense intestinal inflammation leading to diarrhea, weight loss, and gross rectal bleeding, closely modeling the pathological characteristics associated with clinical ulcerative colitis. This model is simple, rapid, easily reproducible, and mimics clinical colitis conditions effectively.

Our data for disease activity index, colonic lipocalin-2 levels, and claudin-4 immunoreactivity showed an effective colitis-like pathophysiology in both the wild type and *App^NL-G-F^* mice. There are multiple clinical and animal studies demonstrating the beneficial effects of using probiotics to treat ulcerative colitis [61,62,63,64,65,66], and our data showed that probiotic feeding significantly reduced colitis pathophysiology, as indicated by reduced disease activity index. However, the probiotic was unable to attenuate lipocalin levels in *App^NL-G-F^* mice, and claudin-4, a tight junction protein associated with the colonic epithelial integrity, showed reduced immunoreactivity with probiotic feeding. This suggested that the probiotic therapeutic intervention was less effective in *App^NL-G-F^* mice and that it possibly even promotes gut leakiness. Clearly, intestinal permeability quantitation will be needed in future work to fully characterize the effects of probiotic treatment alone or in concert with DSS. Interestingly, in the presence of an already DSS-compromised epithelial barrier, it has been reported that probiotic intervention is less able to attenuate inflammatory change, supporting the reduced ameliorative ability we observed [67]. In fact, our prior work has already demonstrated increased intestinal permeability in AD transgenic mouse lines, suggesting that this may be the reason for attenuated efficacy in the *App^NL-G-F^* mice [68]. Our results suggest that it may be necessary to rely on prolonged prophylactic rather than therapeutic probiotic intervention to obtain ideal protective benefits. In addition, varied dosing strategies might be necessary to improve the benefits of probiotic intervention.

Several reports have suggested that the gut–brain axis and gut microbial composition act as significant influencing factors in age-related neurological disorders such as Alzheimer’s disease [18,69,70,71], particularly through their influences on associated neuroinflammatory pathways [72,73]. We observed an unexpected immune-related change in the brain following both DSS and probiotic treatment in wild type mice, which manifested increased neutrophil elastase immunoreactivity. Neutrophils are the most abundant leukocytes in the circulatory system, playing a crucial role in various inflammatory responses. They are unique as they have short half-lives of 6–8 h, respond rapidly, and have the ability to capture pathogens with their neutrophil extracellular traps (NETs), which are protruding structures consisting of decondensed chromatin and antimicrobial/granular proteins that enable them to capture and neutralize foreign bodies [74]. The role of neutrophils in the brains of AD patients is still emerging, and recent findings demonstrate that these cells may regulate neuroinflammation associated with AD, possibly contributing to disease [74,75,76]. We observed not only distinct cell staining but also found NET-like structures in the substantia innominate and hypothalamus, suggesting the occurrence of netosis, particularly in the hypothalamus. To our knowledge, this pattern of immunoreactivity is novel and may indicate an unrecognized role for neutrophils in these brain regions in mediating cell death and inflammation, perhaps in response to bacterial products communicating to this region of the brain. It has been shown that the intestinal absorption of the bacterial cell wall component peptidoglycan results in enhanced targeting to the brain [77], fully supporting the notion that gut-derived bacterial products from probiotic-treated *App^NL-G-F^* or colitic mice may contribute to inflammatory changes in the brain. For example, the substantia innominata contains the nucleus basalis of Meynert, a brain region responsible for producing the acetylcholine (Ach) that is used by the cortex and amygdala [78]. The degeneration of these neurons is an aspect of cell loss observed in AD [46,75]. In the central nervous system (CNS), Ach produced by cholinergic neurons acts on α7 nicotinic Ach receptors expressed on microglia and astrocytes, providing a basal reduction in glial activation [79,80,81]. Although further work is required, it is intriguing to consider that the neutrophil-dependent impairment of cholinergic neurons reduces the basal immunomodulatory activity of ACh in the brain, leading to an overall increase in neuroinflammatory phenotype. Indeed, this cholinergic inflammatory reflex is well described for the vagus nerve and central to peripheral immune cell regulation, suggesting that a similar mechanism occurs in the brain [82]. Surprisingly, DSS-induced colitis and probiotic feeding did not produce any significant changes in neutrophil elastase immunoreactivity in the *App^NL-G-F^* mice, suggesting that the AD-associated changes were already maximal, with no additional increase possible due to probiotic intervention or DSS treatment. Further research is needed to determine if bacteria or bacterial products truly increase in the brain during colitis or probiotic intervention in both wild type and *App^NL-G-F^* mice.

Our Aβ plaque and gliosis results were similarly unexpected. DSS-induced colitis combined with probiotic intervention produced a slight increase in plaque immunoreactivity. Consistent with the increase in plaque load, the combined DSS and probiotic treatments elevated microglial reactivity, suggesting that the combination may exacerbate some histologic aspects of disease in mice. Intestinal and neural inflammation in AD mouse models has been previously correlated with an increase in Aβ plaque load in hippocampi during colitis [35,83], perhaps making our results not too surprising. It is known that microglia acquire a reactive phenotype at the site of any trauma during an inflammatory response in the brain, such as those that might occur in the vicinity of Aβ deposits. Many reports regarding both animal models of AD [84,85,86,87,88] and human cases of the disease [89,90,91,92,93,94] have suggested that activated microglia around Aβ plaques demonstrate a tangible interaction with Aβ [95,96]. Reactive gliosis associated with elevated Aβ levels leads to increased levels of pro-inflammatory cytokines such as TNF-α and IFN-γ, which can aggravate an inflammatory response and promote neuronal loss [84,86,97,98,99,100,101,102,103,104,105,106,107,108,109,110]. Therefore, the probiotic intervention was not only seemingly unable to attenuate disease-related changes in the brain but also may have contributed to it. Once again, a possible reason for this effect is that the probiotic bacteria/bacterial products travel from the intestine into the blood before reaching the brain, where they drive a proinflammatory stimulus. As already mentioned, future works that evaluate intestinal permeability as well as the translocation of bacterial products into the brain will help to explain the probiotic potentiation of DSS effects.

Based upon the observations of plaque load, gliosis, and cytokines, we fully expected to see changes in cellular phenotype and elected to use both c-Fos and 5hmC staining to assess this. It is well known that c-Fos is a transcription factor and an immediate early gene that serves as a marker for stimuli-induced changes in brain activity. The expression of c-Fos has a clear correlation with neuron activity [111], as well as glial phenotypes [112,113]. Accordingly, decreases in c-Fos expression correlate with cognitive decline in AD [114,115]. Prior work has demonstrated that manipulating the intestinal microbiome in mice with oral antibiotic reduces brain c-Fos mRNA, which is recovered with probiotic feeding [116]. In fact, in our prior work, we fed probiotics to female *App^NL-G-F^* mice, and this resulted in increased hippocampal c-Fos immunoreactivity [36]. Therefore, we expected to observe differences in c-Fos immunoreactivity with treatment, but it was unanticipated that a robust decrease in both wild type and *App^NL-G-F^* mice would occur due to combined DSS and probiotic treatment. Although we elected to quantify overall brain immunoreactivity in this study, it is possible that a more thorough investigation into selective changes in the brain region or the stereologic quantitation of c-Fos immunoreactivity and overall molecular phenotypes would reveal neuronal differences between treatments. For example, it is not unreasonable to expect that there may be both increases and decreases in staining across brain regions with respect to the same treatment.

Similar to the changes in c-Fos immunoreactivity, analysis of 5hmC staining also revealed interesting changes across treatment groups. DNA methylation is a dynamic process in which methyltransferases (DNMTs) utilize normal DNA cytosine (Cyt) to add a methyl group at the 5-position to generate 5-methylcytosine (5mC), which can oxidize to 5-hydroxymethylcytosine (5hmC) [117]. 5hmC is highly expressed in the brain, and its expression level is especially upregulated during embryonic neurogenesis and also in postnatal life, where it is associated with neural gene expression and activity [118]. It has been reported that 5hmC levels are significantly increased in AD mouse brains when compared with corresponding controls [119]. In our study, 5hmC expression was elevated by probiotic feeding in the wild type group only and increased when combined with DSS, suggesting that epigenetic changes were induced by probiotic feeding. This was entirely consistent with prior reports on the ability of probiotic interventions to induce epigenetic change in the brains of rodents and Zebrafish [120,121]. Interestingly, we also observed changes that resulted from DSS treatment alone in WT mice and no changes at all in the *App^NL-G-F^* mice. One possibility for the lack 5hmC changes in the *App^NL-G-F^* mouse brains is simply that there is a basally elevated amount compared to wild type brains and that it is already at the maximal response. This may indicate the increased expression of numerous genes in the *App^NL-G-F^* mice compared to the wild type mice. Therefore, a more careful analysis of the selective changes in the brain may still reveal the differences among various treatments methods.

## 4. Materials and Methods

### 4.1. Animal Model

*App^NL-G-F^* mice (KI:RBRC06344) were obtained from Dr. Takashi Saito and Dr. Takaomi C. Saido, RIKEN BioResource Center, Japan. These mice carry the humanized Aβ region, including Swedish (NL), Arctic (G), and Beyreuther/Iberian (F) mutations, which promotes Aβ production, enhances Aβ aggregation through facilitating oligomerization and reducing proteolytic degradation, and increases the Aβ_42/40_ ratio, respectively [122]. This transgenic mouse model of AD develops cortical Aβ amyloidosis as early as 2 months. Wild type (WT) C57BL/6 mice were originally purchased from the Jackson Laboratory (Bar Harbor, Maine), and the *App^NL-G-F^* transgenic mice were maintained, as a colony, under standard housing conditions, including a 12 h light/12 h dark cycle and 22 ± 1 °C temperature with access to food and water ad libitum at the University of North Dakota Center for Biomedical Research. Male C57BL/6 control WT and *App^NL-G-F^* mice at 6–10 months of age (n = 7–11 per treatment group) were used. Although sex differences are important to consider, particularly in the context of AD, for this study, we used only males due to their susceptibility to inflammation induced by DSS [123]. The mice were randomly divided into vehicle and DSS-treated groups for 8 weeks of investigation. The mice were euthanized followed by cardiac perfusion, and the brains and colons were collected to quantify the histologic and biochemical changes within. All procedures involving animals were reviewed and approved by the UND Institutional Animal Care and Use Committee (UND IACUC). The investigation conformed to the National Research Council of the National Academies Guide for the Care and Use of Laboratory Animals (11th edition).

### 4.2. DSS Exposure and Probiotic Treatment

*App^NL-G-F^* and WT mice were randomly divided into 4 experimental groups, vehicle (1:1 MediGel and water), 2% DSS, probiotic (Pro), and 2% DSS + probiotic (DSS/Pro). A commercial probiotic, distributed as VSL#3^®^ circa 2016, was procured by the authors and referred to in this study as probiotic; it was comprised of eight strains of lactic acid-producing bacteria: *Lactobacillus plantarum*, *Lactobacillus delbrueckii* subsp. *Bulgaricus*, *Lactobacillus paracasei*, *Lactobacillus acidophilus*, *Bifidobacterium breve*, *Bifidobacterium longum*, *Bifidobacterium infantis*, and *Streptococcus salivarius* subsp. *thermophilus*. The authors understand that this formulation is now available as Visbiome^®^. The probiotic was resuspended in MediGel^®^ (Clear H2O, Portland, ME, USA), and the mice were given either MediGel only or probiotic in MediGel. Probiotic treatment started on day one and lasted for eight weeks. The dose of probiotic (0.32 × 10^9^ CFU bacteria/25 g mice) was calculated based on the body surface area normalization method from the recommended human dose of the probiotic [124]. According to the manufacturer, human colonization takes place over two-three weeks, so the mice were pretreated for three weeks before the DSS treatment [125]. Mice were provided MediGel in water control (vehicle) or MediGel in water containing probiotic *ad libitum* for the entire experimental period, and freshly prepared probiotic or vehicle control was provided every third day. After the probiotic pretreatment period, for the DSS treatment groups, colitis-like disease was induced in two groups by dissolving DSS (2%, *w*/*v*, MW = 36–50 kDa, MP Biomedicals, LLC, Santa Ana, CA, USA) in MediGel in water. A final concentration of 2% DSS was resuspended in diluted MediGel for two cycles, 3 days each, with 14 days of recovery between each exposure as previously described [126]. To calculate the disease activity index, the mice were weighed individually on day 0, every day during the second cycle of the DSS treatment, and 2 days after exposure for the second cycle of DSS administration.

### 4.3. Assessment of the Severity of Colitis-like Symptoms

The disease activity index (DAI) assessment was used to assess colitis severity. It is a compilation of multiple assessments, including the percentage of body weight loss, stool consistency, and fecal blood. A Hemoccult test kit (Beckman Coulter Inc., Brea, CA, USA) was used to determine occult blood in the stool samples according to manufacturer instructions. DAI assessment was conducted in a blinded fashion, starting 1 day prior to DSS treatment (day 0), occurring throughout the second cycle of 2% DSS administration, and continuing until 2 days after DSS exposure, meaning that the assessment ran for a total of 5 days, as previously described [127,128,129,130,131]. Each measurement was scored on a scale ranging from 0 to 4, which was then summed for a DAI score per mouse. A maximum severity score of 12 was possible. To normalize the findings, the daily DAI score per mouse was subtracted from its respective day 0 score. On the 8th week, the animals were euthanized, and their brains and colons were collected for further analysis.

### 4.4. Immunohistochemistry

For all brains, the left hemispheres were fixed in 4% paraformaldehyde for 5 days, followed by cryoprotection through two incubations in 30% sucrose. The hemispheres were then embedded in 15% gelatin and serially sectioned (40 μm) using a sliding microtome [132]. The distal colons were fixed in 4% paraformaldehyde and sectioned (10 µm) onto subbed slides via cryostat. The colon sections were immunostained using antibodies against claudin-4 (1:500, rabbit, ZMD.306; Thermo Fisher Scientific Inc., Waltham, MA, USA). The brain sections were immunostained using antibodies against neutrophil elastase (1:200 dilution, rabbit, ab68672; Abcam, Cambridge, MA, USA), Aβ (1:500 dilution, rabbit, D54D2; Cell Signaling Technology, Inc., Danvers, MA, USA), GFAP (1:1000 dilution, rabbit, D1F4Q; Cell Signaling Technology, Inc., Danvers, MA, USA), Iba-1 (1:1000 dilution, rabbit, 019–19741; Wako Chemicals USA, Inc., Richmond, VA, USA), c-Fos (1:2000 dilution, rabbit, ab222699; Abcam, Cambridge, MA, USA), 5hmC (1:2000, rabbit, ab214728; Abcam, Cambridge, MA, USA) to detect neutrophil elastase, Aβ plaques, astrocytes, microglia, and neuronal activity/phenotype changes, respectively. For elastase, 5hmC, c-Fos, and Aβ, antigen retrieval was required. Neutrophil elastase and 5hmC antigen retrieval were performed using pH 6 sodium citrate at 95 °C for 10 min. c-Fos antigen retrieval was performed using pH 9 tris-EDTA at 95 °C for 10 min. For Aβ antigen retrieval, sections were incubated in 25% formic acid for 25 min at room temperature before blocking. After antigen retrieval, slides (colon) or free-floating sections (brains) were incubated in 0.3% H_2_O_2_ to quench endogenous peroxidases before being rinsed in phosphate-buffered saline (PBS) and blocked in PBS containing 0.5% bovine serum albumin (BSA, Equitech-Bio, Inc., Kerrville, TX, USA), 0.1% Triton X-100 (Sigma-Aldrich, St. Louis, MO, USA), 5% normal goat serum (NGS, Equitech-Bio, Inc., Kerrville, TX, USA), and 0.02% sodium azide for at least 30 min. Slides or free-floating tissue were incubated in primary antibody solution for 24 h at 4 °C before being transferred into biotinylated secondary antibodies, where they were incubated for 2 h at room temperature. After the secondary antibody incubation, a VECTASTAIN Avidin-Biotin Complex (ABC) kit was used followed by the Vector VIP Peroxidase (HRP) Substrate kit (SK-4600) to visualize antibody binding (Vector laboratories, Inc., Burlingame, CA, USA). The colon slides were dehydrated, and the coverslipped and brain sections were mounted onto subbed slides, dehydrated, and coverslipped. The brain slides were imaged and viewed in the Hamamatsu NanoZoomer 2.0HT Brightfield Scanning System.

#### 4.4.1. Quantification of Aβ, GFAP, and Iba-1 Staining

The quantification of Aβ, GFAP, and Iba-1 staining was from 3 serial sections/mouse and was conducted using an open-source digitalized image analysis platform, QuPath (v.0.4.3) [133]. The hippocampus regions of all brain sections were annotated using either the brush tool or wand tool. Staining quantitation was performed as previously described [134]. For each tissue section, QuPath grouped adjacent and similar pixels into a superpixel of 25 mm^2^. Pixel similarity was determined by red-green-blue (RGB) values. After grouping into superpixels, QuPath software was used to apply an arbitrary intensity value for each superpixel to identify whether they were either positive or negative [134]. The % Aβ, GFAP, or Iba-1 positive superpixels calculated by Qupath were averaged and graphed as mean values +/− SEM.

#### 4.4.2. Quantification of Elastase, c-Fos, and 5hmC Staining

Whole slide images of the IHC-stained sections were acquired using Hamamatsu NanoZoomer 2.0-HT slide scanner (Hamamatsu Photonics, Hamamatsu City, Japan) at ×20 magnification. The quantification of neutrophil elastase, 5hmC, and c-Fos from 3 to 10 hemibrain coronal serial sections/mouse was also performed using QuPath (v.0.4.3) [133]. The QuPath workflow for quantification included: creating a project, adding images, setting the image type to DAB, pre-processing using estimate stain vectors, simple tissue detection to detect all tissues on the whole slide, splitting into single annotation, positive cell detection based on optical density, adjusting parameters (maximum area, intensity threshold, cell expansion, selecting cell/nucleus compartments for scoring intensity threshold), the automated counting of positive cells (detections) within a small region, manual verification of positive detections, creating a script from the workflow, running the groovy script for automated quantification across entire brain regions and across all the tissues in the project, and exporting the annotation measurements to Microsoft Excel [133,135,136,137,138]. The % positive cells calculated in QuPath {% Positive = [(Total no. of detections—No. of negative detections)/Total no. of detections] × 100)} was used for data analysis and comparison.

### 4.5. Enzyme-Linked Immunosorbent Assay (ELISA)

On the collection day, the right hemispheres and middle parts of the colons were isolated and flash frozen. The hippocampi, temporal cortices, and middle colons were lysed in RIPA buffer (20 mM Tris, pH 7.4, 150 mM NaCl, 1 mM Na3VO_4_, 10 mM NaF, 1 mM EDTA, 1 mM EGTA, 0.2 mM phenylmethylsulfonyl fluoride, 1% Triton X-100, 0.1% SDS, and 0.5% deoxycholate), lysis buffer 17 (R&D Systems, a Bio-techne brand, Minneapolis, MN, USA), and 1% Triton X-100 in PBS, respectively, with a protease inhibitor cocktail (P8340, Sigma-Aldrich, St. Louis, MO, USA). The tissues were centrifuged (12,000 rpm, 4 °C, 10 min), and the supernatants were collected. The hippocampi supernatants were used to perform soluble Aβ 1-40/42 ELISAs (human Amyloid β 1-40/42 Brain EZBRAIN40/42 ELISA, EMD Millipore, Billerica, MA, USA). The hippocampi pellets were re-suspended in 5M guanidine HCL/50mM Tris HCL, pH 8.0, and centrifuged (12,000 rpm, 4 °C, 10 min), and the supernatants were removed to quantify insoluble Aβ_1–40/42_ levels by using the same ELISA kit according to the manufacturer’s protocol. Temporal cortex lysates were used for cytokine analysis. The colon supernatants were used to perform lipocalin-2 ELISAs (Mouse Lipocalin-2/NGAL DuoSet ELISA, R&D Systems, a Bio-Techne brand, Minneapolis, MN, USA) according to the manufacturer’s protocol. The BCA kit (ThermoFisher Scientific, Rockford, IL, USA) was used to quantify protein concentrations.

### 4.6. Western Blotting

Temporal cortices were lysed in RIPA buffer and spun, and the supernatants were quantified via BCA assay, and five μg of protein per temporal cortex lysate was resolved by 10% sodium dodecyl sulfate polyacrylamide gel electrophoresis (SDS-PAGE) and transferred to polyvinylidene difluoride membranes (PVDF) for Western blotting. Membranes were blocked for 1h in Intercept (TBS) Protein-Free Blocking Buffer (LI-COR Bioscience, Lincoln, NE, USA) followed by incubation with anti-PSD95 (1:000, ab 238135; Abcam, Cambridge, MA, USA) and synaptophysin (1:000, ab52636; Abcam, Cambridge, MA, USA) antibodies overnight at 4 °C. Near-infrared-labeled secondary antibodies were used to detect antibody binding using IRDye^®^, 680RD-, or 800CW-labelled secondary antibodies 1:15,000 (LI-COR Bioscience, Lincoln, NE, USA). Blots were scanned using a LI-COR Odyssey imaging system (LI-COR Bioscience, Lincoln, NE, USA). Band intensity values were normalized to their respective loading control, glyceraldehyde-3-phospate dehydrogenase (GAPDH) (sc32233; Santa Cruz Biotechnology, Santa Cruz, CA, USA). Band intensities were quantified using ImageJ software. A band size was selected that fit the entirety of each band on the blot. The software was used to measure band intensity in each lane, which was normalized to its relevant loading control, GAPDH values, from the same membrane.

### 4.7. Th1/Th2/Th17 Cytokine Assessment

RayBiotech Quantibody^®^ Mouse Th1/Th2/Th17 Q1 arrays (QAM-TH17-1-1, RayBiotech, Norcross, GA) were employed to assay brain cytokine levels from temporal cortex lysates following the manufacturer’s protocol. Eighteen cytokines were evaluated: IL-1β, IL-2, IL-4, IL-5, IL-6, IL-10, IL-12p70, IL-13, IL-17, IL-17F, IL-21, IL-22, IL-23, IL28, IFN-γ, MIP-3α, TGF-β, and TNF-α. Completed arrays were sent to RayBiotech, where a GenePix 4400 scanner was used to scan the slide arrays using GenePix Pro software. The results were analyzed using the RayBiotech Analysis Tool. Using a standard curve, concentrations of each cytokine were determined.

### 4.8. Statistical Analysis

Results are presented as mean values ± standard error mean (SEM). Statistical analysis was performed by one-way ANOVA followed by uncorrected Fisher’s LSD test to determine statistical differences, and DAI, Lipocalin-2 ELISA, and %BW changes were analyzed via two-way ANOVA multiple comparisons followed by uncorrected Fisher’s LSD test or non-parametric one-way ANOVA (Kruskal–Wallis test) followed by Dunn’s multiple comparison test. GraphPad Prism 8 software (GraphPad Prism Inc., La Jolla, CA, USA) was used, with *p* < 0.05 considered statistically significant.

## 5. Conclusions

The probiotic intervention was capable of attenuating the disease activity index associated with the colitis-like condition induced by DSS exposure in both wild type and *App^NL-G-F^* mice. In wild type brains, although the colitis-like condition had minimal effects on glial immunoreactivities or cytokine changes, it did increase neutrophil elastase immunoreactivity and decrease c-Fos staining, suggesting some communication of intestinal inflammation to the brain in these mice. In *App^NL-G-F^* mice, the colitis-like condition did not change neutrophil elastase, glial, or c-Fos staining, although it did alter levels of numerous cytokines in the brain and elevated levels of soluble Aβ 1-40, suggesting that, once again, some aspect of the intestinal inflammation was propagating to the brain. Perhaps the most surprising observation was the effect of probiotic feeding alone on both genotypes of mice. Probiotic treatment alone increased neutrophil elastase staining, decreased c-Fos immunoreactivity, and attenuated PSD95 levels in both wild type and *App^NL-G-F^* mice, demonstrating effects on the brain independent of any concomitant AD or colitis-like phenotype. Moreover, combined probiotic and DSS treatment had a few robust AD-related effects on the *App^NL-G-F^* mice compared to DSS treatment alone, including additional increases or decreases in brain cytokines, potentiation of Aβ plaque immunoreactivity, and increased microgliosis. Collectively, our findings indicate that the effects of DSS-induced colitis, a probiotic diet, and combined probiotic/DSS produce unique and varied effects on the brains of both wild type and *App^NL-G-F^* mice in contrast to fairly congruent effects in the intestine. This variability highlights the complexity of planning a dietary probiotic approach with the intent to mediate specific target changes in the brain. Future works comparing probiotic interventions to more common pharmacologic therapeutics may be useful in determining whether variations in the gut-to-brain communication of inflammatory changes during colitis are reduced.

## Data Availability

All data are included in the published article.
